# κ-Opioid Receptor Agonists as Robust Pain-Modulating Agents: Mechanisms and Therapeutic Potential in Pain Modulation

**DOI:** 10.3390/jcm14207263

**Published:** 2025-10-15

**Authors:** Mario García-Domínguez

**Affiliations:** 1 Program of Immunology and Immunotherapy, CIMA-Universidad de Navarra, 31008 Pamplona, Spain; mgdom@unav.es; 2 Department of Immunology and Immunotherapy, Clínica Universidad de Navarra, 31008 Pamplona, Spain; 3 Centro de Investigación Biomédica en Red de Cáncer (CIBERONC), 28029 Madrid, Spain

**Keywords:** pain, kappa opioid receptor, agonist, analgesia, chronic pain, G-protein biased agonism, neuroimmune interactions, central sensitization

## Abstract

**Background/Objectives**: κ-Opioid receptors have emerged as promising targets for novel analgesic strategies, offering the potential to relieve pain without the adverse effects commonly associated with μ-opioid receptor activation, such as respiratory depression, tolerance, and addiction. This review focuses on recent advances in understanding KOR-mediated pain modulation and aims to evaluate the therapeutic potential of KOR agonists in addressing the limitations of current opioid-based treatments. **Methods**: This review synthesizes evidence from comprehensive preclinical studies investigating the effects of KOR agonists on central pain pathways, including modulation of neurotransmitter release and attenuation of ascending nociceptive signaling. In addition, emerging clinical trial data on KOR-selective compounds will be evaluated, together with recent advances in biased agonism and region-specific receptor signaling, to guide the development of next-generation analgesics. **Results**: Preclinical studies demonstrate robust antinociceptive effects of KOR agonists, while early clinical trials indicate that several KOR-selective compounds effectively reduce pain symptoms. Advances in biased agonism and targeted receptor signaling suggest the potential to achieve analgesia with reduced dysphoria and sedation. **Conclusions**: KOR-targeted therapies show significant translational potential for pain management. The integration of preclinical and clinical evidence supports the development of next-generation KOR agonists that could provide effective analgesia while minimizing the adverse effects associated with conventional opioids.

## 1. Introduction

Pain is defined by the International Association for the Study of Pain (IASP) as “an unpleasant sensory and emotional experience associated with, or resembling that associated with, actual or potential tissue damage” [1,2]. This definition underscores two critical aspects: (1) pain is not merely a physiological signal, but also a subjective experience influenced by emotional, cognitive, and cultural factors [3]; and (2) pain sensation can exist even in the absence of detectable tissue injury, as observed in various chronic pain syndromes [4]. From a biological standpoint, acute pain plays a protective role, serving as a warning system that promotes withdrawal from harmful stimuli and initiates reparative behaviors [5]. In contrast, chronic pain continues beyond the expected duration required for tissue healing (typically >3 months), losing its adaptive value and becoming a pathological condition in its own right [6]. This maladaptive transformation involves structural and functional plasticity at multiple levels of the nervous system, leading to central sensitization, spontaneous firing of nociceptors, and abnormal amplification of sensory input [7,8]. It is estimated that over 20% of adults live with chronic pain, with prevalence increasing with age and comorbid conditions [9,10]. Neuropathic pain (arising from lesions or dysfunction of the somatosensory nervous system) and inflammatory pain (driven by immune-mediated processes) are particularly refractory to standard treatments [11,12].

At the molecular level, pain arises from complex interactions between neuronal and non-neuronal cells. Primary pathways include the following: (1) Ion channel dysfunction: VGSCs (voltage-gated sodium channels, e.g., Nav1.7, Nav1.8, and Nav1.9) and TRPs (transient receptor potential channels, e.g., TRPV1—transient receptor potential vanilloid 1- and TRPA1—transient receptor potential ankyrin 1-) decisively regulate nociceptors [13]. (2) Neuroimmune interactions: Several pro-inflammatory cytokines released by immune cells sensitize nociceptors, sustaining peripheral hyperalgesia. Spinal glia, including astrocytes and microglia, secrete pro-nociceptive mediators that enhance excitatory neurotransmission, contributing to central sensitization [14]. (3) Neurotransmitter and receptor modifications: Central sensitization evokes increased glutamatergic transmission through NMDA (N-methyl-D-aspartate) receptors in the spinal dorsal horn, alongside impaired inhibitory signaling (GABAergic and glycinergic), reducing endogenous pain control [15]. (4) Physiological analgesic mechanisms: The opioid (MOR, DOR, and KOR) and endocannabinoid (CB1 and CB2) systems facilitate endogenous pain relief, but chronic pain leads to downregulation or desensitization, diminishing efficacy and promoting persistent pain [16,17,18,19].

For decades, opioid analgesics (primarily acting on MOR) have represented the gold standard for the management of moderate-to-severe pain [20]. MOR agonists, like morphine, fentanyl, and oxycodone, produce analgesia mainly through Gi/o protein-coupled mechanisms [21]. These pathways inhibit AC (adenylyl cyclase) activity, decrease cAMP (cyclic adenosine monophosphate) levels, activate GIRK (G protein-coupled inwardly rectifying potassium channel) channels, and suppress VGCCs (voltage-gated calcium channels), collectively decreasing neuronal excitability and attenuating neurotransmitter release within pain pathways [22,23]. However, prolonged MOR stimulation induces profound neuroadaptations, such as upregulation of AC, receptor desensitization, and altered dopaminergic signaling in mesolimbic circuits, which contribute to the development of tolerance, dependence, and addiction [24,25,26]. Simultaneously, MOR agonists carry life-threatening risks, most notably respiratory depression mediated through brainstem circuits, and contribute directly to the escalating global opioid epidemic [27]. These pharmacological limitations highlight an urgent demand for alternative opioid receptor targets capable of providing effective analgesia while minimizing the deleterious effects associated with MOR activation.

Within this context, the KOR has emerged as an especially attractive candidate. KOR, alongside other members of the opioid receptor family, is a seven-transmembrane GPCR (G-protein-coupled receptor) chiefly coupled to Gi/o proteins [28]. It is widely expressed across CNS (central nervous system) and PNS (peripheral nervous system) structures implicated in nociceptive processing, including the DRG (dorsal root ganglion), spinal dorsal horn, PAG (periaqueductal gray), amygdala, and nucleus accumbens [29,30]. Following activation (by endogenous ligands such as dynorphins A and B or α/β-neoendorphins, as well as by pharmacological agonists) [31,32], KOR triggers several intracellular cascades, inhibition of AC, suppression of VGCCs, and activation of GIRK channels, collectively reducing excitatory neurotransmitter release (such as glutamate, substance P, and CGRP—calcitonin gene-related peptide) at spinal and supraspinal sites, thus dampening ascending nociceptive signaling [33,34,35]. KOR activation potentiates descending inhibitory pathways through noradrenergic and serotonergic signaling, contributing to analgesia [36,37].

Preclinical studies consistently indicate that KOR agonists exert robust analgesic effects across a range of nociceptive, inflammatory, and neuropathic pain models. Systemic administration of KOR agonists reduces behavioral hypersensitivity to thermal, mechanical, and chemical noxious stimuli; the effects are mediated not only via central modulation of dorsal horn excitability, but also via peripheral actions on nociceptors, where KORs inhibit hyperexcitability and neuropeptide release in inflamed tissue [38,39,40,41]. Importantly, unlike MOR agonists, KOR activation does not interact with the mesolimbic dopaminergic system to the same extent, thus producing little to no euphoria or reinforcement, a property that substantially lowers the abuse potential of these compounds [42].

Despite these advantages, the therapeutic development of KOR agonists has historically been blocked by centrally mediated adverse effects [43]. Activation of KORs in mesolimbic and limbic circuits, particularly the nucleus accumbens and amygdala, is associated with dysphoria, anhedonia, and psychotomimetic effects [44]. The clinical utility of first-generation KOR agonists has been impeded by central side effects [45]. Recent pharmacological advances are addressing these limitations, with peripherally restricted KOR agonists delivering potent analgesia without triggering central side effects [46]. Similarly, G protein-biased KOR ligands, currently in preclinical or clinical development, retain strong analgesic efficacy while exhibiting enhanced tolerability [47].

Collectively, the convergence of preclinical data, clinical validation, and advances in receptor pharmacology positions KORs as highly promising targets for next-generation analgesics, suggesting that historical limitations of KOR agonists can potentially be overcome to fully harness their therapeutic potential in pain management.

This review seeks to present a comprehensive synthesis of KOR-mediated pain modulation, encompassing molecular mechanisms, preclinical evidence, and data from clinical trials. Moreover, recent pharmacological advances are highlighted, and their implications are examined for the development of next-generation KOR agonists designed to provide effective analgesia with reduced adverse effects. Ultimately, the exploration of KOR-targeted therapies represents a promising avenue for safer pain control.

To address this, a comprehensive search of PubMed, MEDLINE, EMBASE, and the Cochrane Library was conducted using the following keywords: *κ-opioid receptor agonists*, *kappa opioid receptor*, *pain modulation*, *analgesia*, *pain management*, *mechanism of action*, *therapeutic potential*, *opioid receptors*, *preclinical studies*, and *clinical studies*. Studies were initially screened by title and abstract, followed by full-text review to determine eligibility. Exclusion criteria included publications prior to 1985, non-English studies, and studies lacking data on the variables summarized in the tables.

## 2. Insights into κ-Opioid Receptors and Their Role in Pain Modulation

As a dynorphin-activated GPCR, the KOR plays essential roles in orchestrating nociceptive signaling, stress responses, emotional regulation, and autonomic control. Its study requires integrating two complementary perspectives, including anatomical mapping of receptor distribution across the human body and characterization of intracellular signaling cascades that link ligand binding to biological processes. Taken together, these aspects elucidate both the therapeutic promise of the receptor and its contribution to maladaptive physiological and behavioral outcomes.

### 2.1. Spatial Expression Patterns Within the CNS and PNS

KORs, members of the GPCR superfamily, exhibit extensive distribution across both the CNS and PNS, with region-specific expression profiles that underpin their functional roles in nociceptive modulation, stress responsivity, affective regulation, and autonomic control [29,48].

Within CNS regions, KOR expression is elevated in the hypothalamic paraventricular and arcuate nuclei, PAG, dorsal horn, amygdaloid complex, bed nucleus of the stria terminalis, and mesolimbic structures (including VTA-ventral tegmental area- and nucleus accumbens) [49,50,51,52]. Moreover, KORs are strongly expressed in the ACC (anterior cingulate cortex), insula, putamen, neocortex, caudate nucleus, thalamus, globus pallidus, pons, substantia nigra, and hippocampus [53]. Importantly, KOR expression is not confined to neuronal populations; glial cells also express KORs, thus participating in neuromodulatory processes and neuroimmune signaling [54].

In the PNS, KORs are widely expressed in primary afferent nociceptors, sympathetic and parasympathetic ganglia, and within visceral sensory pathways, specifically those innervating the gastrointestinal tract and urinary bladder [29,55,56]. KORs are also detected in peripheral organs, like the heart [57], lungs [58], kidneys [59], and reproductive organs [60,61], where they participate in many homeostatic functions. Finally, KORs are also expressed in some immune cells, such as T and B lymphocytes, macrophages, and dendritic cells, suggesting an essential role in modulating neuroimmune interactions, inflammatory responses, and peripheral immune signaling [54,62,63].

### 2.2. Molecular Cascades Triggered by KOR Stimulation

KOR activation stimulates a multifaceted intracellular network, integrating Gi/o protein-coupled signaling with β-arrestin-mediated noncanonical pathways, both of which exert temporally and spatially distinct effects on cellular physiology, including regulation of receptor desensitization and downstream kinase activation [64]. Following ligand binding of endogenous ligands, principally dynorphins, KOR undergoes conformational rearrangements within its transmembrane helices and intracellular loops, thereby facilitating their biological activity [65].

Dissociation of Gαi/o from Gβγ subunits yields parallel signaling outputs: (1) inhibition of AC, leading to a reduction in cAMP levels and downstream suppression of PKA (protein kinase A) activity, which profoundly impacts phosphorylation states of CREB (cAMP response element-binding protein) and other transcriptional regulators [66,67]; (2) regulation of ion channel activity, wherein Gβγ subunits inhibit N- and P/Q-type VGCCs, reducing neurotransmitter vesicle release, while concomitantly activating GIRK channels, thus hyperpolarizing neuronal membranes and attenuating excitability [68,69].

**Figure 1:**
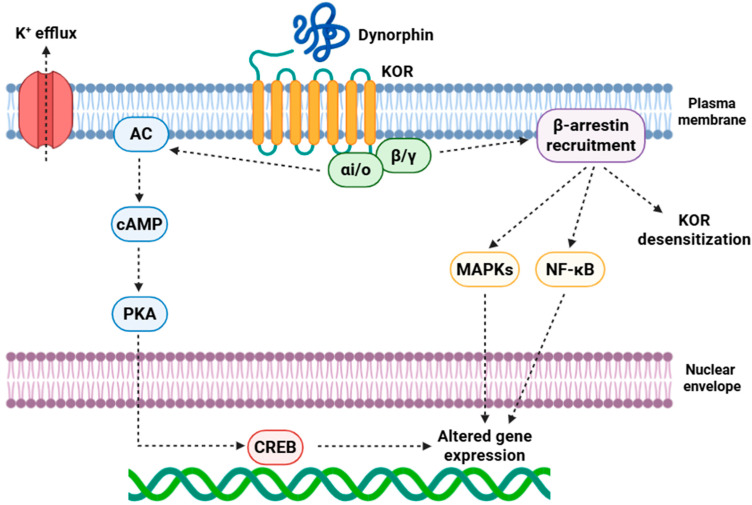
Dynorphin activates KOR, suppressing AC activity through Gαi/o activity to decrease cAMP/PKA signaling and CREB-dependent transcription, while Gβγ promotes K^+^ efflux through GIRK channels to decrease neuronal excitability. Concurrent β-arrestin recruitment drives receptor desensitization and scaffolds MAPK and NF-κB pathways, collectively modulating gene expression and mediating long-term cellular adaptations. Abbreviations: KOR (κ-opioid receptor), Gi (G-protein, inhibitory alpha subunit), Go (G-protein alpha subunit o), K^+^ (potassium ion), Gβγ (G-protein beta-gamma subunits), AC (adenylyl cyclase), cAMP (cyclic adenosine monophosphate), PKA (protein kinase A), CREB (cAMP response element-binding protein), MAPK (mitogen-activated protein kinase), and NF-κB (nuclear factor kappa-light-chain-enhancer of activated B cells). Image created with BioRender.

Beyond these canonical mechanisms, receptor phosphorylation by GRKs (G protein-coupled receptor kinases) allows for β-arrestin recruitment, specially β-arrestin2, which orchestrates receptor desensitization, internalization through clathrin-coated pits, and scaffolding of kinase modules [70]. β-arrestin-mediated pathways robustly activate MAPKs (mitogen-activated protein kinases), including ERK1/2 (extracellular signal-regulated kinases 1/2) and p38 MAPK, with the latter mechanistically implicated in KOR-induced dysphoria, stress responsivity, and maladaptive synaptic plasticity [71]. On the other hand, KOR signaling intersects with PI3K (phosphoinositide 3-kinase)/Akt (protein kinase B) cascades and modulates NF-κB (nuclear factor kappa-light-chain-enhancer of activated B cells) signaling, influencing gene expression (Figure 1) [72].

### 2.3. Neurobiological Roles in Pain Processing

#### 2.3.1. Inhibition of Excitatory Neurotransmitter Release

**Figure 2:**
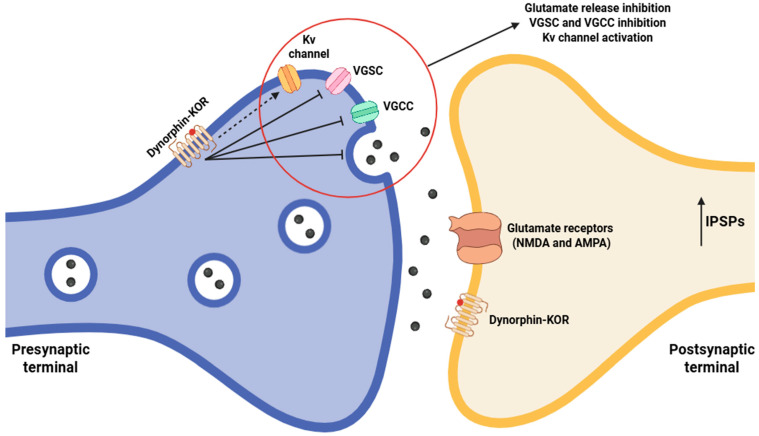
Schematic of dynorphin-mediated modulation in the spinal cord, involving both pre- and postsynaptic mechanisms. Presynaptic KOR activation promotes Kv channels opening and VGSC and VGCC inhibition, leading to hyperpolarization and reduced excitatory neurotransmitter release, including glutamate. Combined with postsynaptic KOR activation, this generates IPSPs, suppresses action potential firing, and attenuates ascending nociceptive signaling. Abbreviations: KOR (κ-opioid receptor), Kv (voltage-gated potassium channel), VGSC (voltage-gated sodium channel), VGCC (voltage-gated calcium channel), NMDA (N-methyl-D-aspartate receptor), AMPA (α-amino-3-hydroxy-5-methyl-4-isoxazolepropionic acid receptor), and IPSP (inhibitory postsynaptic potential). Diagram created with BioRender.

Activation of KORs initiates a complex intracellular signaling cascade that mediates a pronounced modulatory effect on nociceptive neurotransmission. The reduction in intracellular Ca^2+^ that follows KOR activation critically disrupts the Ca^2+^-dependent exocytotic machinery underlying neurotransmitter release in nociceptive primary afferent terminals [73]. Following activation, KORs reduce Ca^2+^ influx through N-type and P/Q-type VGCCs, thereby diminishing the local Ca^2+^ microdomains required for vesicle fusion [74]. Ca^2+^ binds to synaptotagmin, a low-affinity, fast-responding Ca^2+^ sensor embedded in the vesicle membrane, which induces conformational changes that allow for the SNARE (soluble NSF attachment protein receptor) complex (formed by VAMP—vesicle-associated membrane protein-/synaptobrevin, syntaxin, and SNAP-25—synaptosomal-associated protein of 25 kDa-) to transition from a partially zippered to a fully zippered state, culminating in membrane fusion and neurotransmitter exocytosis [75]. Insufficient Ca^2+^ binding prevents synaptotagmin from facilitating the conversion of the SNARE complex into a fusion-ready configuration, resulting in slowed vesicle fusion kinetics and a decreased likelihood of neurotransmitter release [76]. This mechanism preferentially affects synchronous, action potential-triggered release of excitatory neurotransmitters such as glutamate and neuropeptides, including substance P, CGRP, and NPY (neuropeptide Y), which are fundamental for nociceptive signaling (Figure 2) [77,78].

Beyond the Ca^2+^-dependent pathway, Gβγ subunits directly modulate the vesicular fusion machinery. Gβγ interacts with SNARE components, exerting steric hindrance and allosteric modulation. These interactions disrupt the precise alignment and zippering of the SNARE complex, effectively immobilizing vesicles in a docked but fusion-incompetent state [79]. Additionally, Gβγ can recruit presynaptic accessory proteins such as complexin, which further stabilizes the partially primed SNARE complex and imposes a block on spontaneous or evoked vesicle fusion [80]. This dual inhibition provides that neurotransmitter release is suppressed even in the presence of residual Ca^2+^ influx.

Concurrently, KOR activation encourages the opening of GIRK channels [81]. GIRK facilitate K^+^ efflux, hyperpolarizing the presynaptic membrane. Hyperpolarization raises the voltage threshold required for activation of VGSCs, thereby reducing action potential amplitude and reducing the propagation velocity along C- and Aδ-fiber nociceptors [82]. By diminishing presynaptic excitability, this mechanism synergizes with the suppression of Ca^2+^ influx to further lower the probability of synaptic vesicle fusion, providing a powerful, multimodal inhibitory effect on excitatory neurotransmission.

Ultrastructural analyses using immunogold electron microscopy provide further insight into the precise subcellular localization of KORs. These studies reveal that KORs are predominantly situated on presynaptic membranes immediately adjacent to active zones, the specialized sites within presynaptic terminals where synaptic vesicles are organized and primed for exocytotic release [73]. The proximity of KORs to docked vesicles positions them optimally to regulate key molecular events governing neurotransmitter release, including vesicle tethering, SNARE complex assembly, and Ca^2+^-dependent fusion. Moreover, KORs reside in close proximity to VGCCs, allowing for the inhibition of Ca^2+^ entry and concurrent Gβγ-mediated modulation of SNARE-dependent vesicle release [83,84]. Immunohistochemical studies demonstrate a dense distribution of KOR immunoreactivity along the presynaptic terminals of these primary afferents, indicating that KORs are enriched at sites critically involved in the initial stages of synaptic transmission [29,85].

Finally, KORs exhibit a highly restricted yet functionally strategic pattern of expression within the superficial dorsal horn of the spinal cord, predominantly in laminae I and II [86]. These laminae represent the termination zones for nociceptive afferents, including both C-fibers and myelinated Aδ-fibers, which convey noxious thermal, mechanical, and chemical stimuli arising from peripheral tissues [87]. This postsynaptic localization suggests that KORs may be involved in modulatory circuits, including feedback and feedforward inhibition, potentially shaping the excitatory-inhibitory balance within dorsal horn circuits [88,89].

#### 2.3.2. Suppression of Ascending and Descending Nociceptive Transmission

KORs are expressed throughout the CNS and are strategically localized to exert potent inhibitory modulation of nociceptive processing across numerous anatomical levels, thereby regulating not only the initial transduction of peripheral noxious stimuli, but also their ascending transmission through spinal and supraspinal pathways, their integration within limbic and autonomic networks, and their final representation in cortical regions mediating conscious pain perception and higher-order cognitive emotions [90].

At the spinal level, lamina I contains projection neurons that give rise to the principal ascending nociceptive pathways, such as the spinothalamic and spinoparabrachial tracts [91,92], whereas lamina II is composed predominantly of interneurons that regulate the balance of excitatory and inhibitory input converging onto these projection neurons [93]. The activation of KORs suppresses excitatory transmission presynaptically by inhibiting neurotransmitter release from primary afferent terminals [73], and postsynaptically by inducing hyperpolarization of projection neurons, thus reducing the excitability of lamina I outputs and effectively gating nociceptive transmission at its initial central relay [86].

By regulating neuronal activity within the dorsal horn, KORs attenuate the transmission of nociceptive signals through the spinothalamic tract to thalamic relay neurons and their cortical targets, thus reducing the fidelity of the somatotopic representation of painful stimuli [94]. On the other hand, KOR expression within both the spinal origins of the spinoparabrachial tract and within the parabrachial nucleus itself enables suppression of excitatory drive into these circuits, thus reducing the affective salience of pain and its capacity to activate autonomic and homeostatic responses such as cardiovascular and respiratory adjustments [95].

At the midbrain level, KORs within the PAG regulate excitatory input to descending projection neurons, biasing the system toward inhibitory rather than facilitatory output, which potentiates descending inhibitory control at the spinal level [96]. The RVM (rostral ventromedial medulla), which play a key role in modulating spinal nociceptive signals, is controlled by KORs, thus further potentiating descending antinociceptive control [97,98]. In these regions, KOR activation modulates the balance between inhibitory (GABAergic) and excitatory (glutamatergic) inputs onto descending projection neurons [99]. Inhibition of AC and the subsequent reduction in cAMP levels influences neurotransmitter release within these circuits, increasing the activity of inhibitory serotonergic and noradrenergic neurons that descend to the dorsal horn [100].

Finally, KORs are distributed in higher-order neural circuits mediating the affective and cognitive aspects of pain. Within the amygdala, KOR activation decreases excitatory transmission within these nuclei (e.g., central nucleus and basolateral complex), dampening the affective-motivational component of pain [101].

### 2.4. Alterations in KOR Expression and Signaling in Chronic Pain States

Alterations in the expression and functional dynamics of KORs have been increasingly recognized as critical contributors to the complex pathophysiology of chronic pain states [35]. In persistent nociceptive contexts, preclinical and clinical studies have documented region-specific changes in KOR expression, with several areas showing receptor upregulation (possibly as a compensatory mechanism) and others exhibiting downregulation or desensitization, which can impair analgesic signaling [102,103].

Functionally, chronic pain often biases KOR signaling away from canonical Gi/o protein-mediated inhibitory pathways toward β-arrestin-dependent signaling. This shift has been linked to enhanced activation of p38 MAPK in glial populations, contributing to neuroinflammation and sustained pain hypersensitivity [104]. Changes in receptor phosphorylation patterns, internalization rates, and modifications in receptor trafficking further reinforce dysregulation, leading to altered receptor availability at the membrane and impaired signal fidelity [105]. Regarding transcriptional regulation, epigenetic modulation of the OPRK1 gene has been observed in several models of neuropathic and inflammatory pain, indicating that transcriptional reprogramming may underlie chronic pain-induced KOR alterations [106].

The interplay between KOR dysregulation and neuroimmune signaling appears to be bidirectional and highly dynamic. Increased levels of pro-inflammatory cytokines, including IL-1β, have been shown to impair KOR-mediated analgesic signaling by altering receptor expression, desensitization kinetics, and downstream G-protein or β-arrestin-dependent pathways [107]. Alternatively, aberrant KOR activity can influence the release of cytokines and chemokines (e.g., IL-1β, TNF-α, and CXCL1) from neurons and glial cells, thus reinforcing neuroinflammatory signaling and contributing to the maintenance of central and peripheral sensitization [108]. This reciprocal crosstalk suggests that chronic pain-associated changes in KOR function are not merely a downstream consequence of inflammation, but actively participate in shaping the neuroimmune environment, creating a self-perpetuating loop that sustains nociceptive hypersensitivity and may exacerbate the sensory and affective dimensions of chronic pain.

Collectively, the evidence indicates that chronic pain induces a reorganization of the KOR system. This reorganization encompasses alterations in receptor expression levels across specific CNS and PNS sites, shifts in signaling pathway bias promoting β-arrestin-dependent versus canonical Gi/o-mediated pathways, and disruptions in receptor trafficking and membrane localization. Furthermore, transcriptional and epigenetic mechanisms, including modifications of the *OPRK1* gene, seem to play a fundamental role in these persistent adaptations [109]. Such adaptations not only influence nociceptive processing, but also underpin the affective, motivational, and cognitive aspects of persistent pain, emphasizing the function of KORs across sensory and emotional pain circuits. Consequently, therapeutic strategies that target distinct components of KOR signaling hold substantial promise for the development of next-generation, non-addictive analgesics capable of alleviating the sensory and affective burdens of chronic pain.

## 3. Classical KOR Agonists in Pain Modulation

Classical KOR agonists constitute a fundamental pharmacological tool in the investigation of pain pathways and their underlying mechanisms, serving both as experimental tools and as potential therapeutic drugs. Unlike MOR agonists, KOR-selective compounds provide robust antinociceptive effects with a reduced risk of life-threatening side events such as respiratory depression and severe constipation. These pharmacodynamic characteristics underscore classical KOR agonists as promising candidates for the development of safer analgesic modalities and support their continued assessment.

### 3.1. Therapeutic Potential of Classical KOR Agonists in Experimental Pain

Classical KOR agonists have been extensively investigated in preclinical pain models, encompassing several nociceptive, inflammatory, and neuropathic paradigms. KORs have emerged as essential modulators of pain transmission at both spinal and supraspinal levels [86,90]. Early pharmacological studies employed prototypical KOR agonists such as U-50488 and U-69593, revealing analgesic properties across numerous modalities of experimentally induced pain [43].

KOR agonists (Table 1) demonstrate potent analgesic effects across a range of preclinical pain models. In preclinical inflammatory pain models, including those induced by formalin or Complete Freund’s Adjuvant (CFA), KOR agonists showed potent efficacy in thermal and mechanical nociceptive assays [110,111,112,113,114,115,116,117,118,119,120,121]. KOR agonists also exhibit efficacy in several neuropathic pain models, where they suppress mechanical allodynia and thermal hyperalgesia [122,123,124,125,126,127]. Finally, emerging evidence further supports the analgesic potential of KOR agonists in preclinical cancer pain models. In rodent models of tumor-induced bone cancer pain, KOR agonists palliate nociceptive behaviors, highlighting their capacity to target pain mechanisms distinct from traditional MOR agonists [128,129,130,131].

### 3.2. Human Trials of KOR-Selective Compounds

Several KOR agonists have advanced into clinical trials for the treatment of pain (Table 2). Although numerous preclinical studies demonstrate robust antinociceptive effects and promising efficacy in models of pain, pruritis, and inflammation [110,111,112,113,114,115,116,117,118,119,120,121,122,123,124,125,126,127,128,129,130,131], these results have not reliably translated into clinically approved analgesics, in part due to several adverse effects (e.g., aversion, dysphoria, sedation, and mood disturbances) that limit dosing in humans [45]. Furthermore, current clinical trials face additional limitations, including small sample sizes, short treatment durations, and inadequate pharmacokinetic and pharmacodynamic characterization, which hinder the interpretation of efficacy and tolerability outcomes [132,133]. Moreover, the adverse psychotomimetic and dysphoric effects of acting KOR agonists have restricted dose escalation and clinical viability [134]. Finally, translational medicine is limited by interspecies differences in receptor pharmacology, behavioral endpoints, and metabolic pathways, as well as by the inherent challenges of faithfully recapitulating the complexity of human pain states in preclinical models. Systematic recognition and mitigation of these translational barriers are essential to improve the predictive validity of preclinical research and to facilitate successful clinical translation [135].

HSK21542 is being evaluated in phase 2 and phase 3 clinical studies, demonstrating strong analgesic efficacy and good tolerability in patients subjected to abdominal surgery [136,137]. Difelikefalin (CR845) is recently approved for the treatment of pruritus in patients receiving hemodialysis [138] and has also showed dose-dependent antinociceptive effects in a phase 2 clinical trial in osteoarthritis [NCT02524197] as well as in patients with notalgia paresthetica [139]. In a phase 2 trial, ADL 10-0101 produced a significant reduction in persistent visceral pain scores following intravenous infusion [140]. Correspondingly, asimadoline (EMD-61753) improved abdominal pain, increased pain-free days, and enhanced overall symptom relief in randomized, double-blind trials and phase 2 studies of patients with irritable bowel syndrome (IBS) [141,142]. A subsequent phase 2 study in patients with diarrhea-predominant IBS (IBS-D) confirmed its efficacy, demonstrating improvements in abdominal pain, urgency, and stool frequency [NCT00955994]. Fedotozine, assessed in IBS patients, increased colonic distension thresholds while maintaining normal compliance [143]. CR665, tested in a randomized, double-blind trial, elevated esophageal pain thresholds, but reduced tolerance to cutaneous pain [144]. Finally, apadoline (RP 60180), evaluated in healthy volunteers, attenuated cortical responses to painful nasal CO_2_ stimulation, as evidenced by a 40% reduction in pain-related cortical potentials [145].

### 3.3. Safety and Tolerability Compared with MOR Agonists

The safety profile of classical KOR agonists represents a key differentiating feature compared to MOR agonists [32]. Preclinical and clinical evidence consistently indicates minimal risk of respiratory depression, reduced incidence of constipation, and low abuse potential, rendering KOR-targeted therapies particularly attractive for populations vulnerable to opioid-related adverse events [45].

Nevertheless, KOR agonists exhibit a distinct spectrum of side effects. Centrally acting compounds can induce dysphoria, anhedonia, sedation, and, in some cases, psychotomimetic effects including hallucinations [146]. Dysphoria appears to be dose-dependent and is correlated with CNS penetration [147], motivating the development of peripherally restricted or G-protein-biased KOR agonists designed to preferentially activate analgesic pathways while limiting β-arrestin-mediated signaling linked to negative affect [148]. Importantly, KOR activation is intricately involved in the regulation of stress responsivity, primarily via modulation of the hypothalamic–pituitary–adrenal (HPA) axis and corticotropin-releasing factor (CRF) systems [149]. Prolonged stress contributes to KOR activation, exacerbating stress-related neuroendocrine responses and promoting negative emotional states [150]. Moreover, accumulating evidence suggests that sustained or high-level KOR activation can potentiate depressive-like and anxiogenic behaviors, thereby posing a potential risk for worsening mood disorders in susceptible individuals [151].

In contrast, chronic activation of KORs may lead to receptor desensitization and tolerance in preclinical models, while these adaptations emerge more slowly than with MOR agonists [152]. Moreover, KOR agonists modulate endocrine function, principally prolactin and stress-axis hormones, needing monitoring during administration [153].

Overall, the evidence supports classical KOR agonists as a promising analgesic alternative to conventional opioids. Key challenges include optimizing analgesic efficacy while minimizing central side effects, improving pharmacokinetic profiles for chronic use, and addressing interindividual variability in human responses. Advances in biased agonism, peripheral restriction, and combination strategies are expected to enhance the clinical applicability of KOR-targeted therapies in the coming decade.

## 4. Recent Advances in KOR Agonist Development

Recent years have witnessed notable progress in KOR agonist development, driven by advances in understanding receptor pharmacology, signaling pathway selectivity, and tissue-specific receptor distribution. Traditional KOR-targeted therapies have been limited by adverse neuropsychiatric effects, such as dysphoria and sedation, which arise primarily from non-selective activation of intracellular signaling pathways. Contemporary research has focused on rational ligand design and precision-targeted delivery strategies to overcome these limitations, emphasizing the exploitation of biased agonism, receptor micro-switch modulation, and region-specific drug localization. These approaches aim to maximize therapeutic benefits, while minimizing off-target and β-arrestin2-mediated adverse outcomes, thereby establishing a new framework for the clinical translation of KOR-directed interventions.

### 4.1. Biased Agonism and Pathway-Selective Signaling

Recent investigations into KOR pharmacology have unveiled the remarkable therapeutic potential of biased agonism, a paradigm in which ligands selectively stabilize distinct receptor conformations that preferentially engage specific intracellular signaling cascades [154]. High-resolution structural and biophysical analysis, like cryo-EM (cryogenic electron microscopy) and NMR (nuclear magnetic resonance) analyses, have shown that KOR ligands potentiate conformational changes within essential transmembrane helices, particularly TM3, TM5, and TM6 [70]. These conformational rearrangements preferentially orient the receptor toward Gi/o protein coupling rather than β-arrestin2 recruitment, thereby enabling selective intracellular signaling [155]. Engagement of Gi/o proteins results in strong inhibition of AC, a consequent reduction in cAMP levels, and modulation of downstream effectors, such as MAPK signaling cascades, phospholipase C, and GIRK channels [28]. These signaling events enable robust analgesic and anti-pruritic responses while bypassing β-arrestin2-dependent pathways, which are typically linked to dysphoria, sedation, motor deficits, and other adverse neuropsychiatric effects [71].

Mechanistic investigations integrating computational docking, molecular dynamics simulations, and structure–activity relationship (SAR) analyses have elucidated the molecular basis by which these ligands induce biased signaling. Complementary approaches, such as site-directed mutagenesis, have further identified specific residues that are critical determinants of this signaling bias [156,157]. To illustrate, the residue D138^3.32^, located in TM3, plays a crucial role in KOR ligand recognition and signaling bias [158]. Its negatively charged carboxylate side chain establishes a highly conserved ionic interaction with the protonated amine groups of the ligands, thus ensuring precise positioning of the ligand within the orthosteric site [159]. This electrostatic contact drives conformational stabilization of TM3 and its associated microswitches, which propagates to the intracellular receptor interface, favoring Gi/o protein coupling over β-arrestin2 recruitment. Molecular dynamics simulations suggest that perturbation of this interaction, either through ligand modifications or site-directed mutagenesis, can shift the receptor conformational landscape toward alternative states, highlighting its role in dictating functional selectivity [160]. In a similar fashion, Y320^7.43^, situated in TM7, forms part of an intricate hydrogen-bonding network that connects the orthosteric pocket to the intracellular face of the receptor [158]. The hydroxyl group can form polar interactions with adjacent residues and water molecules, allosterically influencing the conformation and dynamic behavior of the intracellular loops (ICL2 and ICL3), which are crucial for β-arrestin2 recruitment. Through modulation of the rotameric conformations of TM7 and the spatial organization of the cytoplasmic termini of TM3-TM6, Y320^7.43^ indirectly regulates the signaling bias of the receptor [159]. Ligand-specific interactions with this residue can stabilize receptor conformations that preferentially recruit Gi/o proteins or, conversely, enhance β-arrestin2 association, providing a molecular explanation for the functional selectivity observed among different KOR agonists [160].

Numerous pharmacological agents exemplify the concept of G protein-biased KOR agonism, providing critical insight into the therapeutic potential of functional selectivity [161]. Classical synthetic agonists, like U-50488 and U-69593, have long been characterized as preferential activators of Gi/o-mediated signaling [162,163]. Although biased toward G protein activation, U-50488 and U-69593 retain partial recruitment of β-arrestin2 [164,165], which can contribute to moderate side effects such as mild sedation or dysphoria in preclinical behavioral models [166,167]. Recent advances in the development of highly G protein-biased agonists have enabled the pharmacological separation of therapeutic benefits from adverse β-arrestin2-mediated effects. Clinically, nalfurafine, approved in Japan and Korea for the treatment of pruritus [168], promotes Gi/o-mediated signaling with minimal β-arrestin2 activation and analgesic and antipruritic effects without inducing appreciable sedation, dysphoria, or motor impairment [169,170]. Several synthetic derivatives, including RB-64, HS665, and some salvinorin analogs, demonstrate similarly pronounced G protein bias, emphasizing the potential of chemical modifications to fine-tune receptor signaling [171,172,173]. Small-molecule scaffolds, including triazole-based compounds, tetrahydroisoquinoline derivatives, and triazole-pyridine hybrids such as KSC-12-192, have been engineered to preferentially activate Gi/o signaling over β-arrestin2 recruitment [174].

Finally, parallel approaches have examined allosteric modulation to enhance the precision of receptor signaling [175]. BMS-986187, a positive allosteric modulator (PAM) of KOR, acts as an ago-PAM, capable of activating the receptor independently of orthosteric ligands while promoting G-protein signaling over β-arrestin2 recruitment [176].

### 4.2. Region-Specific Targeting and Delivery Strategies

In parallel with rational ligand design, the development of region-specific targeting strategies has emerged as an essential paradigm to optimize both the therapeutic efficacy and safety profile of KOR-directed interventions [177]. KORs are expressed across diverse regions of the CNS and maintain significant representation within peripheral tissues, reflecting their involvement in central and systemic physiological processes [29,30], and indiscriminate activation of this receptor is linked to deleterious off-target effects [71]. These liabilities have historically limited the clinical utility of KOR agonists despite their potential analgesic, antipruritic, and anti-addictive properties.

In response to these limitations, considerable progress has been made in engineering advanced delivery modalities that enable regionally restricted activation of the receptor. Lipid- and polymer-based nanoparticles, such as PEGylated liposomes carrying U50,488H [178], have been shown to accumulate in several neuroanatomical substrates, such as the spinal dorsal horn and the PAG. In parallel, U50,488 and salvinorin analogs [179,180], undergo site-specific enzymatic bioconversion within the CSF (cerebrospinal fluid) or spinal cord, thereby restricting the release of active KOR agonists to targeted neuroanatomical regions. Receptor-targeted conjugates, including KOR-selective peptide-drug conjugate ligands, establish regionally restricted drug release and precise receptor activation [181]. Such spatial precision allows for the selective potentiation of Gi/o protein-coupled signaling cascades in neural circuits mediating analgesia while minimizing β-arrestin2 recruitment in regions implicated in aversive or dysphoric behavioral responses [182].

Beyond pharmacological delivery systems, emerging neurotechnological approaches provide additional layers of precision. Chemogenetic approaches, exemplified by the application of designer receptors exclusively activated by designer drugs (DREADDs), enable conditional and reversible activation of KOR-related signaling pathways in genetically defined neuronal populations [183]. Ultimately, viral vector-mediated approaches enable precise, cell type- and circuit-specific modulation of KOR expression, signaling bias, and associated downstream transcriptional cascades [184]. By selectively manipulating receptor density and functional coupling within defined neuronal populations, this approach allows for the dissection of KOR-mediated circuit dynamics and behavioral outcomes, providing a powerful platform for both mechanistic studies and the development of highly targeted therapeutic interventions [185].

## 5. Challenges and Future Perspectives

With the clinical development of KOR agonists, it is crucial to evaluate the obstacles that may impede their broad adoption and the potential avenues for their integration into modern pain management paradigms. Effective translation requires a comprehensive understanding of pharmacological constraints, interpatient variability, and the prospects for combinatorial strategies that can optimize analgesic efficacy while minimizing adverse outcomes.

### 5.1. Clinical Translation and Integration into Pain Management Practice

Despite the robust preclinical evidence supporting the analgesic efficacy of KOR agonists, their clinical translation remains challenging [186]. Early-generation KOR agonists demonstrated potent analgesic effects; however, their widespread use was limited by central CNS-mediated side effects, such as dysphoria, sedation, psychotomimetic symptoms, cognitive impairment, dizziness, fatigue, and mood disturbances [45]. Additional peripheral effects comprise diuresis, nausea, gastrointestinal discomfort, and decreased gastrointestinal motility, which in sum limit their therapeutic applicability [32]. These adverse effects have hindered the clinical translation of KOR-targeted therapies, preventing their integration into routine pain management [187]. This barrier is highly evident in chronic pain settings, where long-term tolerability and patient adherence are critical, and where the adverse effects of KOR agonists pose substantial barriers to sustained therapeutic use [188].

Advances in pharmacological strategies, such as the development of biased agonists that preferentially activate G-protein signaling over β-arrestin pathways, peripherally restricted KOR agonists, and selective allosteric modulators, constitute encouraging options to overcome these limitations [148,175,189]. These strategies are designed to maintain robust analgesic efficacy while attenuating CNS-mediated adverse effects, thereby enhancing the safety profile and supporting the feasibility of long-term therapeutic administration. Despite this, comprehensive clinical evaluation is indispensable to validate these approaches, necessitating evaluation of dose–response profiles, long-term safety and tolerability, receptor desensitization mechanisms, and pharmacological interactions with conventional analgesics or adjunct therapies.

Incorporation into pain management strategies also requires careful consideration of patient heterogeneity, encompassing comorbid conditions, polypharmacy, and variability in receptor expression profiles [190,191]. Individualized therapeutic strategies, guided by biomarkers of KOR function or pain phenotype, might be critical to optimizing therapeutic outcomes and lowering side events [192]. Moreover, regulatory guidance and clinician education will play essential roles in facilitating the adoption of KOR-targeted interventions into clinical practice [193,194].

### 5.2. Potential Synergy with Other Analgesic Approaches

KOR agonists may also provide enhanced clinical utility when employed in combination with other analgesic modalities. Preclinical studies indicate that KOR agonists can act synergistically with MOR agonists, or adjuvant therapies, facilitating lower doses of each agent while achieving effective pain control (Table 3) [39,125,195].

Moreover, KOR agonists may complement with non-pharmacological interventions, including neuromodulation, physical therapy, and cognitive-behavioral strategies, by targeting distinct pain pathways [196,197,198,199]. Their ability to modulate sensory and affective dimensions of pain further supports their inclusion in pain management strategies, particularly in chronic pain states where single-agent therapies provide insufficient relief [44].

Future research should focus on systematically evaluating combination regimens in both preclinical models and clinical trials, with attention to pharmacodynamic interactions, optimal timing, and individualized treatment protocols. Identification of synergistic pairings could accelerate clinical translation and establish KOR-targeted therapies as integral components of comprehensive, multimodal pain management frameworks.

## 6. Conclusions

KOR agonists constitute a promising class of analgesics with unique pharmacological and mechanistic profiles. Preclinical and clinical data demonstrated efficacy in inflammatory, neuropathic, and cancer-associated pain, with lower risks of respiratory depression, tolerance, dependence, and abuse compared to MOR agonists. Mechanistically, KOR agonists modulate GPCR signaling, suppress hyperexcitable spinal circuits, inhibit peripheral inflammatory mediators, and enhance descending inhibitory pathways. G protein-biased agonism over β-arrestin signaling further improves analgesic efficacy while minimizing CNS adverse effects. Structural and pharmacological insights facilitate the rational design of next-generation agonists with enhanced selectivity and tolerability.

Clinical translation is constrained by CNS adverse effects associated with early-generation compounds. However, recent advances in peripherally restricted agonists, G protein-biased ligands, and allosteric modulators offer strategies to maximize analgesic efficacy while reducing CNS side effects. Successful clinical implementation requires an integrated translational framework that aligns molecular pharmacology with regulatory science and patient-centered strategies [200]. Regulatory approaches should account for the distinct pharmacodynamic and pharmacokinetic properties of biased and peripherally restricted KOR agonists, with rigorous evaluation of off-target effects, long-term safety, and abuse potential. Collaboration with regulatory authorities, adaptive trial designs, and biomarker-based endpoints may enable efficient yet robust development pathways [201].

From a clinical and patient-oriented perspective, KOR-targeted therapies should integrate patient-centered outcomes and personalized dosing to enhance clinical relevance. Precision medicine approaches incorporating genetic, metabolic, and psychosocial stratification may further optimize therapeutic response [202]. Peripherally restricted KOR agonists may serve as adjuncts to conventional analgesics in inflammatory and cancer pain, whereas G protein-biased ligands might be preferred for chronic neuropathic pain to enhance efficacy and minimize CNS adverse effects. These strategies have the potential to reduce opioid reliance, mitigate associated complications, and establish a safer, more versatile analgesic paradigm [203].

Ongoing investigation into KOR biology is expected to deepen our understanding of pain neurophysiology and may transform analgesic therapy, providing effective options for patients who are inadequately managed by current treatments.

## Figures and Tables

**Table 1:** KOR agonists employed in the treatment of numerous preclinical pain models. Abbreviations: CFA (Complete Freund’s Adjuvant), CRD (colorectal distension), NSAID (non-steroidal anti-inflammatory drug), CNS (central nervous system), RVM (rostral ventromedial medulla), TRK-820 (nalfurafine), KOR (kappa opioid receptor), DOR (delta opioid receptor), EMD-61753 (asimadoline), HSK21542 (anrikefon), i.v. (intravenous injection), s.c. (subcutaneous injection), CIPN (chemotherapy-induced peripheral neuropathy), SNL (spinal nerve ligation), BCP (bone cancer pain), and IL-10 (interleukin-10).

Pain Type	Pain Model	Compound	Effects	References
Inflammatory pain	CFA	Dynorphin A (1-7)	Dynorphin 1-7 administration significantly increased paw pressure	[110]
	CRD-induced visceral pain	U-50488	Combined with the NSAID flurbiprofen axetil, U50,488 elicited potent visceral antinociception without CNS side effects	[111]
	CFA	U-69593	RVM infusion of U-69593 attenuated CFA-evoked thermal and tactile allodynia	[112]
	Formalin test	TRK-820 (Nalfurafine)	ICI-199441 and TRK-820 produced analgesic effects in the formalin test (much potent TRK-820 than ICI-199441)	[113]
		ICI-199441		
	Formalin test	MP-1104	MP-1104 reduced pain-like behaviors in both phases of the formalin test, effects abolished by KOR (norbinaltorphimine) and DOR (naltrindole) antagonists	[114]
	Adjuvant- induced arthritis	PNU-50488H	Both drugs had equally powerful anti-inflammatory effects	[115]
		EMD-61753 (Asimadoline)		
	Formalin test	GR-94839	GR-94839 produced antinociception in inflammatory pain at doses with minimal CNS effects	[116]
	Writhing test	HSK21542 (Anrikefon)	HSK21542 dose-dependently inhibited the acetic acid-induced writhing response	[117]
	Formalin test Acetic acid- induced visceral pain	Conorphin-66	Conorphin-66 elicited potent antinociception with minimal CNS effects	[118]
	Formalin test	FE200041	FE200041 exhibited robust antinociception in vivo after i.v. and s.c. administration in the formalin test	[119]
	Acetic acid- induced visceral pain	Fedotozine	Fedotozine and PD-117,302 attenuated visceral hypersensitivity	[120]
		PD-117,302		
	CRD-induced visceral pain	Helianorphin-19	Helianorphin-19 produced peripheral analgesia in CRD-induced visceral pain model without eliciting sedative impairments	[121]
	Formalin test CFA	CAV1001	CAV1001 exhibited significant antinociceptive activity in formalin- and CFA-induced pain models	[122]
Neuropathic pain	(CIPN) Paclitaxel	U-50488 and three analogues	All KOR agonists effectively reversed paclitaxel- induced neuropathic pain without the development of tolerance	[123]
	Unilateral peripheral mononeuropathy	U-69593	Antinociceptive effects evoked by U-69593	[124]
	SNL	BRL-52537	Antiallodynic effects in SNL rats	[125]
	(CIPN) Paclitaxel	MP-1104	MP1104 reduced mechanical and cold allodynia	[126]
	SNL	EMD-61753 (Asimadoline)	Both drugs dose-dependently reduced tactile allodynia	[127]
		ICI-204448		
	(CIPN) Oxaliplatin	LOR17	LOR17 significantly attenuated thermal hypersensitivity	[128]
Cancer pain	BCP	U-50488	U-50488 inhibited BCP without affecting tumor-induced bone loss or tumor progression	[129]
			U-50488 exhibited antihyperalgesic effects in a BCP model	[130]
		GR-89696	GR-89696 (in combination with IL-10) attenuated nociception	[131]
		CAV1001	Both drugs demonstrated efficacy against BCP	[122]
		ICI-204448		

**Table 2:** Therapeutic potential of several KOR agonists: evidence from human trials. Abbreviations: CR845 (difelikefalin), EMD-61753 (asimadoline), IBS (irritable bowel syndrome), IBS-D (diarrhea-predominant irritable bowel syndrome), RP 60180 (apadoline), CO_2_ (carbon dioxide), and CSSEP (chemo-somatosensory event-related potential).

Compound	Clinical Status	Main Indication	Effects	References
HSK21542	Phase 2 clinical trial	Postoperative pain	HSK21542 demonstrated a safety and tolerability profile comparable to placebo across all dose regimens in patients undergoing laparoscopic abdominal surgery	[136]
	Phase 3 clinical trial	Postoperative pain	HSK21542 provided strong analgesia with good tolerability in postoperative abdominal surgery patients	[137]
CR845 (Difelikefalin)	Approved	Pruritus	Effective for treating moderate-to-severe pruritus in adults with chronic kidney disease undergoing hemodialysis	[138]
	Phase 2 clinical trial	Osteoarthritis	Oral CR845 provided dose-dependent pain relief in osteoarthritis patients	[NCT02524197]
		Notalgia paresthetica	Oral difelikefalin produced modestly greater reductions in itch intensity compared with placebo over 8 weeks	[139]
ADL 10-0101	Phase 2 clinical trial	Persistent visceral pain	ADL 10-0101 reduced pain scores from 63 ± 7.6 at baseline to 23 ± 15 at 4 h post-infusion	[140]
EMD-61753 (Asimadoline)	Randomized double-blind and randomized trial	IBS-related abdominal pain	In IBS-D patients with moderate baseline pain, asimadoline significantly improved pain relief, pain scores, pain-free days, urgency, and stool frequency	[141]
	Phase 2 clinical trial	IBS-related abdominal pain	In a 12-week trial of 596 IBS patients, asimadoline improved pain scores, increased pain-free days, and provided adequate relief of pain and discomfort	[142]
		IBS-related abdominal pain	In IBS-D patients with moderate baseline pain, asimadoline showed significant improvements in pain scores, pain-free days, and urgency and stool frequency	[NCT00955994]
Fedotozine	Randomized double-blind and randomized trial	IBS-related abdominal pain	Fedotozine raised colonic distension thresholds in IBS patients without affecting compliance	[143]
CR665	Randomized double-blind and randomized trial	Persistent visceral pain	Compared with placebo, CR665 elevated the pain threshold to esophageal distension while lowering tolerance to cutaneous pinch pain	[144]
RP 60180 (Apadoline)	The study was conducted in healthy volunteers. Pain was elicited using brief pulses of gaseous CO_2_ applied to the nasal mucosa. CSSEPs and subjective pain ratings were recorded in response to these stimuli. RP 60180 reduced pain-related CSSERP amplitudes by 40%			[145]

**Table 3:** Therapeutic potential of several KOR agonists: evidence from human trials. Abbreviations: CRD (colorectal distension), MOR (µ-opioid receptor), KOR (kappa opioid receptor), NCP (17-cyclopropylmethyl-3,14β-dihydroxy-4,5α-epoxy-6β-{[4′-(2′-cyanopyridyl)]carboxamido}morphinan), SNL (spinal nerve ligation), VGCC (voltage-gated calcium channel), P2X3 (purinergic receptor P2X, ligand-gated ion channel, 3), P2X7 (purinergic receptor P2X, ligand-gated ion channel, 7), and i.t. (intrathecal injection).

Pain Type	Pain Model	Compounds	Effects	References
Inflammatory pain	CRD-induced visceral pain	Fentanyl (MOR agonist) Spiradoline (KOR agonist)	Each agent produced complete antinociception when administered alone, while co-administration of fentanyl and spiradoline yielded additive effects at low doses and supra-additive interactions at higher doses	[195]
	Formalin test	NCP (dual MOR and KOR agonist)	NCP exerts potent KOR-mediated analgesia with constipation as the only reported side effect	[39]
Neuropathic pain	SNL	BRL52537 (KOR agonist) Pregabalin (VGCC inhibitor) AF-353 (P2X3 receptor antagonist) A804598 (P2X7 receptor antagonist)	I.t. combination of BRL52537, pregabalin, AF-353, and A804598 synergistically or additively attenuated allodynia evoked by SNL	[125]

## Data Availability

No new data were created or analyzed in this study.

## Abbreviations

The following abbreviations are used in this manuscript:

AC	Adenylyl cyclase
ACC	Anterior cingulate cortex
AMPA	α-amino-3-hydroxy-5-methyl-4-isoxazolepropionic acid
BCP	Bone cancer pain
Ca^2+^	Calcium ion
cAMP	Cyclic adenosine monophosphate
CB1	Cannabinoid receptor type 1
CB2	Cannabinoid receptor type 2
CFA	Complete Freund’s Adjuvant
CGRP	Calcitonin gene-related peptide
CIPN	Chemotherapy-induced peripheral neuropathy
CNS	Central nervous system
CO_2_	Carbon dioxide
CRF	Corticotropin-releasing factor
CR845	Difelikefalin
CRD	Colorectal distension
CREB	cAMP response element-binding protein
cryo-EM	Cryogenic electron microscopy
CSF	Cerebrospinal fluid
CSSEP	Chemo-somatosensory event-related potential
DOR	Delta opioid receptor
DREADD	Designer receptors exclusively activated by designer drug
DRG	Dorsal root ganglion
EMBASE	Excerpta medica dataBASE
EMD-61753	Asimadolíne
ERK1/2	Extracellular signal-regulated kinases 1/2
GABA	Gamma-aminobutyric acid
Gi	G-protein, inhibitory alpha subunit
GIRK	G protein-coupled inwardly rectifying potassium channel
Go	G-protein alpha subunit o
GPCR	G-protein-coupled receptor
GRK	G protein-coupled receptor kinase
Gβγ	G-protein beta-gamma subunits
HPA	Hypothalamic–pituitary–adrenal
HSK21542	Anrikefon
i.t.	Intrathecal injection
i.v.	Intravenous injection
IASP	International Association for the Study of Pain
IBS	Irritable bowel syndrome
IBS-D	Diarrhea-predominant irritable bowel syndrome
ICL2	Intracellular loop 2
ICL3	Intracellular loop 3
IL-10	Interleukin 10
IPSP	Inhibitory postsynaptic potential
K^+^	Potassium ion
KOR	Kappa opioid receptor
Kv	Voltage-gated potassium channel
MAPK	Mitogen-activated protein kinase
MEDLINE	Medical literature analysis and retrieval system online
MOR	Mu opioid receptor
Nav1.7	Voltage-gated sodium channel type 1.7
Nav1.8	Voltage-gated sodium channel type 1.8
Nav1.9	Voltage-gated sodium channel type 1.9
NCP	17-cyclopropylmethyl-3,14β-dihydroxy-4,5α-epoxy-6β-{[4′-(2′-cyanopyridyl)]carboxamido}morphinan
NF-κB	Nuclear factor kappa-light-chain-enhancer of activated B cells
NMDA	N-methyl-D-aspartate
NMR	Nuclear magnetic resonance
NPY	Neuropeptide Y
NSAID	Non-steroidal anti-inflammatory drug
P2X3	Purinergic receptor P2X, ligand-gated ion channel, 3
P2X7	Purinergic receptor P2X, ligand-gated ion channel, 7
p38 MAPK	p38 mitogen-activated protein kinase
PAG	Periaqueductal gray
PEG	Polyethylene glycol
PI3K	Phosphoinositide 3-kinase
PKA	Protein kinase A
PKB/Akt	Protein kinase B
PNS	Peripheral nervous system
RP 60180	Apadoline
RVM	Rostral ventromedial medulla
s.c.	Subcutaneous injection
SAR	Structure-activity relationship
SNAP-25	Synaptosomal-associated protein of 25 kDa
SNARE	Soluble NSF attachment protein receptor
SNL	Spinal nerve ligation
TM3	Transmembrane helix 3
TM5	Transmembrane helix 5
TM6	Transmembrane helix 6
TM7	Transmembrane helix 7
TRK-820	Nalfurafine
TRP	Transient receptor potential
TRPA1	Transient receptor potential ankyrin 1
TRPV1	Transient receptor potential vanilloid 1
VAMP	Vesicle-associated membrane protein (also called synaptobrevin)
VGCC	Voltage-gated calcium channel
VGSC	Voltage-gated sodium channel
VTA	Ventral tegmental area
